# Epigenetic Control of Circadian Clock Operation during Development

**DOI:** 10.1155/2012/845429

**Published:** 2012-03-18

**Authors:** Chengwei Li, Changxia Gong, Shuang Yu, Jianguo Wu, Xiaodong Li

**Affiliations:** National Key Laboratory of Virology, College of Life Sciences, Wuhan University, Luojia Hill, Wuchang District, Hubei, Wuhan 430072, China

## Abstract

The molecular players of circadian clock oscillation have been identified and extensively characterized. The epigenetic mechanisms behind the circadian gene expression control has also been recently studied, although there are still details to be illucidated. In this review, we briefly summarize the current understanding of the mammalian clock. We also provide evidence for the lack of circadian oscillation in particular cell types. As the circadian clock has intimate interaction with the various cellular functions in different type of cells, it must have plasticity and specicity in its operation within different epigenetic environments. The lack of circadian oscillation in certain cells provide an unique opportunity to study the required epigenetic environment in the cell that permit circadian oscillation and to idenfify key influencing factors for proper clock function. How epigenetic mechansims, including DNA methylaiton and chromatin modifications, participate in control of clock oscillation still awaits future studies at the genomic scale.

## 1. Introduction

Mammals have overt circadian rhythms in their physiology and behavior, orchestrated by the suprachiasmatic nucleus of the anterior hypothalamus [1, 2]. The endogenous circadian clock enables organisms to anticipate the regular daily changes in the environment and temporally organize their life activities [3, 4]. Fundamentally, circadian timing functions exist at the cellular level not only for suprachiasmatic neurons, but also for cells of various peripheral tissues [5–9]. 

## 2. A Brief Overview of Clockwork Mechanisms

The past two decades witnessed the rapid pace in gaining in-depth understanding of mammalian clockwork operation. Circadian oscillations are generated at the molecular level by a set of clock genes [10–12]. The mapping and cloning of the *Clock^Δ19^* mutation through ENU mutagenesis and positional cloning set the stage for elucidation of mammalian clockwork mechanisms [13–15]. BMAL1 was soon identified to be the dimerization partner of CLOCK to drive clock gene expression [16, 17]. Mouse *Per* genes were also identified and found to be driven by the CLOCK/BMAL1 dimer [18–20]. CRY1 and CRY2 were later found to have essential roles in the integrity of the circadian clock through inhibiting CLOCK/BMAL1-mediated transcription activation [21, 22]. Thus CLOCK and BMAL1 form the positive limb, while CRY and PER proteins form the negative limb of the transcriptional feedback loop [23]. Later on, more details were elucidated and revisions were made for the clockwork model, including the antagonistic regulations of *Bmal1* transcription by REV-ERB*α* and RORa [24–26], additional clock genes such as *Npas2* and *Bmal2*, and posttranscriptional regulation of clock genes' transcripts [27–29]. CRYs interact with the CLOCK/BMAL1 dimer through interactions with PER proteins [30]. Clock proteins are subject to post-translational modifications that affect their stability, cellular localization, and transcriptional activities [31, 32]. Several kinases were shown to phosphorylate PER proteins [33–38]. Defects in PER phosphorylation have been linked with changes in circadian period [33, 39, 40], although the mechanisms responsible for the observed phenotypes are still not unambiguous [34, 41–44]. Nevertheless, post-translational modifications, in controlling subcellular localization, interaction, and stability of clock proteins, are essential for the delay between transcriptional activation and feedback inhibition that is critical to robust circadian oscillation [30, 45–48]. The clockwork components can also be influenced by multiple cellular signaling pathways to adjust the phases of the running clock [49–58]. Operation of the clockwork can impose temporal expression on the transcriptome with gene expression levels peaking at defined times over the day [59, 60]. The daily interactions between transcriptional activators and inhibitors of the clockwork and their corresponding cis regulatory elements on target genes [12] can achieve sophisticated phase control over gene expression rhythms [48, 61].

## 3. Epigenetics: Circadian Rhythms of Gene Expression

Eukaryotic genomic DNA is packaged around histone proteins into repeating nucleosome units, which further form higher-order chromatin structures [62]. Histones are subject to various modifications and chromatin is remodeled according to cellular needs [63–66]. The so-called “histone-code” [67, 68], or more appropriately “histone language” [69, 70], of various histone modifications and their combinations influences gene transcription activities. CpG methylations within DNA sequences affect histone modifications and nucleosomal organization to impact gene transcription [71, 72]. Current evidence indicates that DNA methylation and histone modifications are intimately linked [73–75].

DNA methylation, histone modifications, and chromatin remodeling are the cornerstones of epigenetics. Epigenetics was originally defined as “the study of mitotically and/or meiotically heritable changes in gene function that cannot be explained by changes in DNA sequence” [76]. A more generalized definition was recently proposed “to avoid the constraints imposed by stringently requiring heritability”: epigenetic events are “the structural adaptation of chromosomal regions so as to register, signal or perpetuate altered activity states” [76]. According to this definition, the daily changes of gene expression driven by the cellular clock and the clock's response to external input all fall into the realm of epigenetics [77]. The core clock genes such as CLOCK/BMAL1 and PERs/CRYs are classified as the positive and negative regulators of the clockwork, respectively. CLOCK possesses intrinsic HAT activity [78]. Biochemical characterization of protein complexes containing clock proteins has found additional proteins involved in histone modifications [79–83]. Changes in histone modifications are also associated with rhythmic expression of clock genes [82, 84]. In the thoroughly studied case of circadian *Dbp* expression, daily changes in histone modifications and nucleosome packing accompanied rhythmic CLOCK/BMAL1 bindings to E boxes within the *Dbp* locus [85–87]. Thus it appears that the circadian clock exerts transcriptional regulation through mechanisms involving histone modifications, although there is still much to be learned, due to the complexity of transcriptional control [88–90]. Intimate interactions also exist between the clockwork and the cellular metabolism [91–94]. Metabolism coupling to the clockwork is also through mediators such as SIRT1, PARP-1, and REV-ERB*α*-NCOR1-HDAC3, that act directly or indirectly through chromatin modification mechanisms [95–99]. CLOCK/BMAL1's binding to the E-box elements and presumably their transcriptional activities are regulated by their post-translational modifications rather than directly by their abundance [32, 45, 87, 96]. CRYs and PERs seem to destabilize/disrupt CLOCK/BMAL1 binding to E-boxes [87], with post-translational modifications in PER2 affect its inhibitory effect on CLOCK/BMAL1 transcriptional activation [30, 95]. It should be noted that different E-box elements within their genomic context seem to differ in their affinities of CLOCK/BMAL1 binding, and direct CRY1 recruitment by CLOCK/BMAL1 to the E-box element within *Dbp* promoter (but not intragenic E-boxes of *Dbp*) has been demonstrated [100]. While circadian changes in DNA methylation in the promoters of clock genes or clock-controlled genes have not been reported to our knowledge, epigenetic inactivation of clock genes due to promoter DNA methylation has been reported in various cancer cells [101]. Specifically in the nervous system, methyl-CpG-binding protein 2 (MeCP2) could be phosphorylated due to neuronal activity and phosphorylation relieves MeCP2's binding to methylated DNA and the inhibition of target gene's of transcription [102]. Light signaling to the central clock in the SCN has been shown to cause MeCP2 phosphorylation [103], in addition to inducing histone modifications that parallel the induction of immediate early genes [104].

## 4. Are Cellular Clocks Ubiquitous? The Case during Development

Most adult tissues have endogenous clocks. Peripheral tissues are typically derived from distinct lineages during development and are specialized for distinct functions. The clockwork must intersect with the unique cellular environments in different types of cells [105]. There are a few exceptions to the omnipresence of the circadian clocks in adult tissues. In the nervous system, few brain regions other than the SCN were shown to have endogenous circadian oscillations [106]. Cells during spermatogenesis have been shown to have clock genes' expression patterns not consistent with clock oscillation [107–109]. Similar observations were also made in immature T cells in the thymus [110]. It is a challenge to characterize the cellular environments that are permissive to circadian oscillation in specific types of cells.

 The oscillation status of the cellular clock during development is largely unclear [111]. Downregulation of clock genes' transcripts (possibly of maternal origin) was seen after fertilization in the zygote [112], without apparent zygotic activation of endogenous expression [113]. In mouse embryonic stem cells, imaging studies at the single-cell level also failed to detect circadian oscillations (which appear after differentiation of the ES cells) [114, 115]. Although clock genes' transcripts could be detected in the conceptus and various mouse fetal tissues [111], circadian rhythmicity in their abundance was rarely detected [116]. Adult-like rhythmic patterns in their expressions were detected only during postnatal development [117]. Paracrine mechanism has been postulated to account for intrinsic synchrony at the tissue level in adult mice [118]. Such mechanisms, while their existence still debatable [119, 120], apparently did not play a role in fetal tissues, which lacked overt circadian rhythmicities at the tissue level [116]. Peripheral clocks of adult tissues are influenced by signals from the central clock [8, 120, 121]. The central clock exerts control over peripheral clocks through neural [122], hormonal [123], systemic cues as a result of feeding control [123–125], and daily body temperature changes [58, 126]. Different tissues may respond differentially to those cues, and those cues may also have complex actions toward the same tissue under different feeding conditions [119, 127, 128]. Fetal tissues most likely were under the influence of rhythmic maternal cues. It is peculiar that clock genes, while obviously expressed, did not seem to form the transcriptional/translation feedback loop in the fetal liver [116, 117]. It is possible that fetal clocks were oscillating at the cellular level but were not synchronized/entrained by maternal rhythms. However, it is also well known that fetal tissues such as the liver clearly differed in differentiation state and metabolic activities from the adult liver [129]. Those functional differences are likely accompanied with transcriptome and epigenomic differences between fetal and adult livers, as cellular epigenetic profiles change during development [72, 130, 131]. Thus fetal mouse liver might possess a unique epigenomic environment that was not permissive to cellular circadian oscillation [105]. Furthermore, the cellular redox ratio ([NAD+]/[NADH]) is known to be very low in the fetal rat liver [132], a situation potentially leading to compromised cellular oscillation due to limited SIRT1 function [95]. It should be noted that in other mammals, such as the primates, the cellular clocks might oscillate in the fetal liver [133].

Development of the circadian timing function is independent of maternal rhythms and resilient to perturbations [134, 135]. However, the clock during ontogeny was suggested to be entrained by maternal rhythms [136–139]. The central clock in the SCN has been shown to oscillate soon after neurogenesis before birth [140]. It could also be affected by exogenous agents such as D1 receptor agonist and melatonin [141, 142]. However, the entrainment mechanisms are largely unknown. For example, D1 receptor agonist is known to induce *c-fos* in the fetal SCN, but it has not been documented whether prenatal D1 agonist treatment led to induction of core clock genes [143]. Melatonin, on the other hand, did not seem to act by induction of immediate-early genes in the fetal SCN [144]. Molecular oscillations of clock gene expression in the suprachiasmatic nucleus were typically weak before birth [145, 146]. The perinatal period is accompanied by changes in hormonal milieu that could trigger epigenetic changes in certain genes [147–149]. We recently found perinatal changes in methylation status of the *mPer1* promoter in the suprachiasmatic nucleus [150]. The significance of this change to clock operation and phase resetting remained to be fully elucidated.

## 5. Perspective

The circadian clock is an essential component of cellular functions in various adult tissues. In fetal tissues and ES cells, such oscillation might not operate. However, previous studies often analyzed daily changes in transcripts' abundance to probe the oscillation status of the clock. Few studies addressed the relative abundance of those transcripts' and their protein products' stoichiometry and posttranslational modifications. The circadian clock is resistant to large fluctuations in overall transcription rates [151]. The clock can also tolerate changes in some components' expression patterns [30, 152–154]. However, rigorous requirements are imposed on the expression rhythms of some clock genes [30, 48, 155]. Future studies should investigate the subcellular distribution and chromatin association of clock genes' products in fetal tissues and ES cells to get a more comprehensive picture of the operation status of the clockwork therein. The epigenomic environment of the fetal tissues and ES cells should also be investigated to address their unpermissiveness to clock operation.
